# Quantitative Mapping of NHS Ester–Protein Reactivity Using Native Top-Down Mass Spectrometry

**DOI:** 10.1021/jasms.5c00395

**Published:** 2026-01-28

**Authors:** Jack L. Bennett, Olivia B. Ramsay, Corinne A. Lutomski, Carla Kirschbaum, Carol V. Robinson

**Affiliations:** † Kavli Institute for Nanoscience Discovery, 6396 University of Oxford, Dorothy Crowfoot Hodgkin Building, Oxford OX1 3QU, U.K.; ‡ Department of Chemistry, Physical and Theoretical Chemistry Laboratory, University of Oxford, Oxford OX1 3QZ, U.K.; § Department of Oncology, University of Oxford, Old Road Campus Research Building, Oxford OX3 7DQ, U.K.

**Keywords:** intact proteins, native top-down mass spectrometry, covalent modification, electrophile reactivity, proteoform analysis, NHS esters

## Abstract

Covalent ligands are widely used to label, probe, and modulate proteins, but peptide-centric readouts obscure how modifications colocalize on intact proteoforms. This can limit insight into ligand mechanism, modification stoichiometry, and the architecture of multisite protein conjugates. We present a general native top-down mass spectrometry workflow that quantifies electrophile reactivity directly on intact proteins. Using NHS esters as a model electrophile class, we apply a deconvolution framework to infer differential reactivity at primary amines across promiscuous, multisite modification patterns. The approach preserves full modification connectivity, avoids sample-preparation artifacts associated with denaturation and digestion, and should extend to electrophiles with unknown reactivity. Overall, this framework provides a general platform for designing covalent therapeutics, bioconjugates, and activity-based probes with proteoform-level resolution.

## Graphical abstract

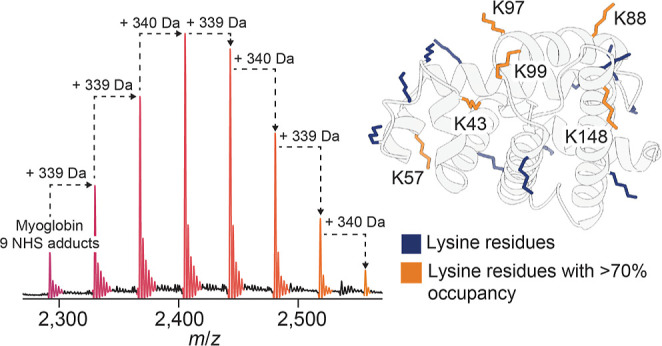

## Introduction

Covalent probes are valuable tools to study and modulate protein function.^1^ In basic research, electrophilic reagents enable attachment of fluorophores, affinity tags, and other labels to facilitate protein detection, purification, and functional characterization.^2–5^ In therapeutic contexts,^6–8^ covalent inhibitors offer distinct pharmacological advantages:^9^ prolonged target engagement, enhanced potency, and in some cases, mutant-selective targeting.^6^ Realizing these benefits, however, requires careful alignment of electrophile reactivity and selectivity with intended outcomes. Undesired or heterogeneous modification can complicate mechanistic interpretation in biochemical studies and drive off-target effects in drug discovery applications.^10^

Mass spectrometry (MS) can greatly accelerate the development of covalent probes with desirable reactivity profiles.^11,12^ Common MS-based approaches for electrophile characterization fall into two categories: (i) peptide-level analysis using bottom-up proteomics,^11,13^ and (ii) intact mass analysis. Peptide-centric analyses can be combined with powerful computational approaches to map electrophile selectivity across the proteome.^14^ However, the requirement for proteolytic digestion prior to analysis results in loss of information regarding concurrent modifications.^15^ Furthermore, denaturation, proteolysis, and other sample preparation steps can compromise quantitative accuracy and induce artifactual side reactions.^16–19^ In contrast, intact protein MS enables quantification of overall stoichiometries with minimal sample manipulation yet lacks the ability to localize individual modifications or quantify site-specific reactivities.^20–23^

Native top-down mass spectrometry (nTDMS) has emerged as a powerful method for protein characterization that combines compositional insights from intact mass analysis with the site-level resolution of bottom-up workflows; its minimal sample preparation also reduces chemical artifacts.^24–28^ Previous work applying denaturing top-down MS to KRAS4B demonstrated that covalent labeling with cysteine-reactive inhibitors can be quantified at the proteoform level and localized to specific sites.^29,30^ However, these studies focused on electrophiles with well-defined target residues and limited possible modification states. It remains to be seen if such approaches could be generalized to study electrophiles with unknown or heterogeneous reactivity, where multiple distinct modification states may coexist.

Here we present a general nTDMS-based workflow for comprehensive analysis of electrophile–protein reactivity. Using multisite NHS ester modification of equine myoglobin as a model system, we develop an unbiased statistical framework that identifies reaction products and localizes individual modification sites while preserving the full connectivity of modification patterns across the intact protein. Our approach is readily applicable to diverse electrophilic chemistries, enabling proteoform-level^31^ characterization of both selective and nonselective covalent probes with previously undefined reactivities.

## Experimental Section

### Materials

All reagents were purchased from commercial suppliers and used without further purification. Fresh biotinamidohexanoic acid *N*-hydroxysuccinimide ester (biotin–NHS), dimethyl sulfoxide (DMSO; Hybri-Max grade), and myoglobin from equine heart were obtained from Sigma-Aldrich. Water (Optima LC–MS grade) was obtained from Fisher Chemical. 10× phosphate-buffered saline was obtained from Invitrogen and diluted with ultrapure water (Milli-Q; Merck Millipore).

### NHS Ester Labeling

Myoglobin (100 μL of 50 μM protein in phosphate-buffered saline, pH 8.0) was incubated with a 25-fold molar excess of biotin–NHS (100 mM stock solution in DMSO) for 15–45 min at 37 °C with gentle agitation. Unreacted biotin-NHS was removed using ultrafiltration (10 kDa molecular weight cutoff; Amicon Ultra, Millipore) by repeated washing (∼1000× reaction volume) with 200 mM ammonium acetate, pH 7.0. The resulting solution was further buffer-exchanged into 200 mM ammonium acetate using gel filtration (7 kDa molecular weight cutoff; Zeba, Thermo Fisher Scientific) and diluted to ∼10 μM for MS analysis.

### Native Top-Down Mass Spectrometry

Analyte solutions were loaded into gold-coated nanoelectrospray ionization (nESI) emitters for MS analysis.^32^ nTDMS measurements were acquired using a hybrid quadrupole–linear ion trap–Orbitrap mass spectrometer^33^ (Orbitrap Ascend Structural Biology Tribrid; Thermo Fisher Scientific) equipped with a static nESI source. Ions were generated by applying a 1.2 kV potential to the nESI emitter relative to the instrument’s inlet capillary, which was maintained at 200 °C. No in-source activation was applied. The instrument was operated in intact protein mode at high pressure (20 mTorr N_2_ in the ion routing multipoles) to maximize transmission of large ions.

MS^1^ spectra were acquired in the Orbitrap with a resolving power of 15,000 at *m*/*z* 200. Automatic gain control (target 4 × 10^5^ charges, maximum inject time 500 ms) was used to minimize space charge effects. Multiple scans were averaged in QualBrowser v.4.5.4747.0 (Thermo Fisher Scientific) following enhanced Fourier transformation of time-domain transients. Data were analyzed using UniDec v.8.0.3.^34^

For MS^2^ analyses, protonated myoglobin ions with 9–14 covalent biotin-NHS adducts (9+ charge state) were isolated using the quadrupole (window size *m*/*z* 5) and activated by higher-energy collisional dissociation (80 V; HCD) in the front high-pressure multipole. We chose to focus on these stoichiometries as they were the major products of the reaction. MS^2^ spectra were acquired in the Orbitrap with a resolving power of 240,000 at *m*/*z* 200. Automatic gain control was again used to minimize space charge effects. For each spectrum, ∼500 time-domain transients were averaged. Spectra were converted to text file format using QualBrowser v.4.5.4747.0 (Thermo Fisher Scientific) and analyzed using precisION v.0.3.0^27^.

### Denaturing Top-Down Mass Spectrometry

The buffer-exchanged myoglobin sample (30 min reaction) was diluted 2× with a mixture of acetonitrile and isopropanol (9:1 v/v) supplemented with 2% formic acid. The resulting solution was analyzed in a similar manner to the native protein samples in intact protein mode at normal pressure. For MS^2^ analyses, protonated *apo* myoglobin ions with 10 covalent biotin-NHS adducts (15+ charge state) were isolated using the quadrupole (window size *m*/*z* 5) and activated by HCD (40 V) in the front high-pressure multipole. All other tuning parameters were maintained between analyses.

### Data Analysis

To confirm the mass of biotin-NHS adducts following collisional activation, data were processed using standard workflows in precisION v.0.3.0.^27^ Putative isotopic envelopes were first detected from deconvolved MS^2^ spectra using DeconTools THRASH v.1.1.7370 ^35,36^ and TopFD v.1.6.4.^37^ A subset of clustered envelopes was manually evaluated and used to train a supervised voting classifier, which was subsequently applied to filter the full set of envelopes. precisION’s fragment-level open search was used to identify sets of sequence ions sharing a common mass offset from canonical *b*- and *y*-type sequence ions. Mass offsets with E-values below 0.001 were considered significant.

To localize and quantify sites of modification, MS^2^ spectra were processed using the proteoform analysis module in precisION. Theoretical isotopic envelopes for myoglobin sequence ions bearing 0–14 biotin–NHS adducts were fit directly to the deconvolved spectra. A mass tolerance of 5 ppm and fitting score threshold of 0.5 was applied to filter potential matches before manual inspection. Sequence ions for which multiple modified forms with consistent charge states could be detected were selected and used to calculate the average number of adducts between a residue (the site of fragmentation) and the N (for *b*-type ions) or C (for *y*-type ions) terminus (Figure S1). For sequence ions with incomplete modification data following automated filtering, spectra were manually reinspected to quantify additional modified sequence ions, and the corresponding missing values were filled accordingly. Values derived from *y*-type ions were converted from the C-terminal reference frame to the N-terminal reference frame by subtracting the average number of adducts assigned to the corresponding *y*-type ion from the total number of adducts measured on the precursor (Figure S2). To account for cases where fragment coverage did not resolve individual reactive residues, lysine residues were grouped into “bins”, where each bin contained one or more residues that could not be distinguished based on the observed fragmentation sites in any of the six spectra. Only lysine residues and the proteins N-terminus were considered reactive, consistent with prior whole-proteome studies of NHS ester reactivity.^14^ Per-bin modification occupancies were estimated by constrained least-squares, enforcing non-negativity and a fixed total adduct count (Supporting Note S1). Uncertainty was assessed using 10,000 residual bootstrap resamples. To obtain a single occupancy estimate for each bin across multiple precursor stoichiometries, we computed a precursor-abundance–weighted average of the stoichiometry-resolved occupancies. Uncertainty distributions for these estimates were obtained by propagating residual bootstrap samples through the abundance-weighted average. A Monte Carlo simulation (10,000 realizations) was used to model intact stoichiometry distributions based on these average occupancies.

### Peptide Mapping with Nanoflow Liquid chromatography–mass Spectrometry

The myoglobin sample (30 min reaction) was prepared for peptide mapping using miniprep-assisted sample preparation.^38^ The protein was reduced using TCEP and alkylated with iodoacetamide, followed by acidification with phosphoric acid. The acidified sample was loaded onto a miniprep column (GeneJET; Thermo Fisher Scientific) and washed four times with 100 mM Tris in 90% methanol. The column-bound protein was digested overnight at 37°C using 2 μg chymotrypsin–standard tryptic cleavage was not used as its efficiency may be altered by lysine modification. Peptides were sequentially eluted with (i) 50 mM Tris, (ii) 0.2% formic acid in water, and (iii) 50% acetonitrile in water. Eluates were combined, dried, and resuspended in 0.1% formic acid in water at a final concentration of 100 ng/μL.

Peptides were separated using nanoflow reversed-phase liquid chromatography (Ultimate 3000, Thermo Fisher Scientific) with a 58 min gradient. Mobile phase A consisted of 0.1% formic acid in water, and mobile phase B consisted of 0.1% formic acid in acetonitrile (v/v; Fisher Scientific, LC–MS grade). Peptides (300 ng, 3 μL injection volume) were loaded onto a 5 mm × 300 μm trap cartridge packed with 5 μm C18 beads (pore size 100 Å; Acclaim PepMap, Thermo Fisher Scientific) and washed with 100 μL mobile phase A before separation on a 15 cm × 75 μm analytical column packed with 3 μm C18 beads (100 Å pore size; Acclaim PepMap). Peptides were separated at 300 nL/min and 25 °C using the following gradient: 5–40% B over 40 min, 40–99% B over 5 min, 99% B for 5 min, followed by re-equilibration at 5% B for 8 min.

Eluting peptides were infused into a hybrid quadrupole–linear ion trap–Orbitrap mass spectrometer (Orbitrap Eclipse Tribrid, Thermo Fisher Scientific) via a stainless-steel nanobore emitter (Thermo Fisher Scientific). Tuning parameters included a capillary voltage of 2400 V, capillary temperature of 320 °C, and RF amplitude of 60%. Data were acquired in data-dependent acquisition mode with a 3 s cycle time. Survey scans were acquired in the Orbitrap over an *m*/*z* range of 300–2000 with quadrupole isolation enabled, a resolving power of 120,000 at *m*/*z* 200, and an AGC target of 4 × 10^5^ charges. Precursor ions with charge states 2–5 and intensities greater than 5 × 10^4^ were selected for fragmentation, with dynamic exclusion enabled after one occurrence for 30 s, including isotopes. Precursors were isolated using a ±0.5 Th quadrupole window and fragmented by HCD with 30% normalized collision energy. MS/MS spectra were acquired in the Orbitrap with an automatic scan range, a resolving power of 30,000 at *m*/*z* 200, and an AGC target of 4 × 10^5^ charges.

Proteomics data were searched using MSFragger v.4.3.^39^ Searches were performed against a database containing the mature equine myoglobin sequence supplemented with common contaminants and reverse decoy sequences (downloaded December 2025). Precursor and fragment mass tolerances were set to 20 ppm, and the isotope error window was set to 0/1/2/3. Mass calibration and parameter optimization were enabled. Enzymatic specificity was set to “chymotrypsin”, allowing up to two missed cleavages. Carbamidomethylation of cysteine was specified as a fixed modification, while methionine oxidation, protein N-terminal acetylation, and biotin–NHS labeling of lysine and protein N-termini were included as variable modifications (maximum of four variable modifications per peptide). Peptide–spectrum matches were validated using PeptideProphet,^40^ and protein inference was performed using ProteinProphet.^41^ Peptide- and protein-level false discovery rates were sequentially filtered to 1% using Philosopher v.5.1.2.^42^ Label-free quantification (LFQ) was performed using IonQuant v.1.11.11.^43^ After filtering the final data set contained 1162 peptide–spectrum matches, 774 of which were assigned to equine myoglobin, corresponding to 24 unique peptides. Site-specific occupancies were calculated from IonQuant intensities using a custom Python script. Extracted ion chromatograms were generated using QualBrowser v.4.5.474.0 (Thermo Fisher Scientific).

## Results and Discussion

### NHS Ester Labeling of Equine Myoglobin

To generate a multisite protein bioconjugate typical of many electrophile chemistries, we incubated equine myoglobin with a 25-fold molar excess of biotin–NHS for 15–45 min (Figure S3) and analyzed the resulting product using native MS (Figure 1a). In the MS^1^ spectrum, after a 30 min reaction, we observed a single predominant charge state distribution between *m*/*z* 2000–3000, which we assigned to differentially modified heme-bound myoglobin (21,640 Da vs 21,638 Da theoretical mass) with between 9–16 biotin–NHS adducts. Adjacent sets of peaks were separated by ∼339 Da, corresponding to the expected increase in mass following reaction between the labeling reagent and a primary amine (339.16166 Da; Figure 1b). Additional finely spaced peaks within each adducted species were also observed; we assigned these to sodium adducts (∼22 Da spacing).

**Figure 1:**
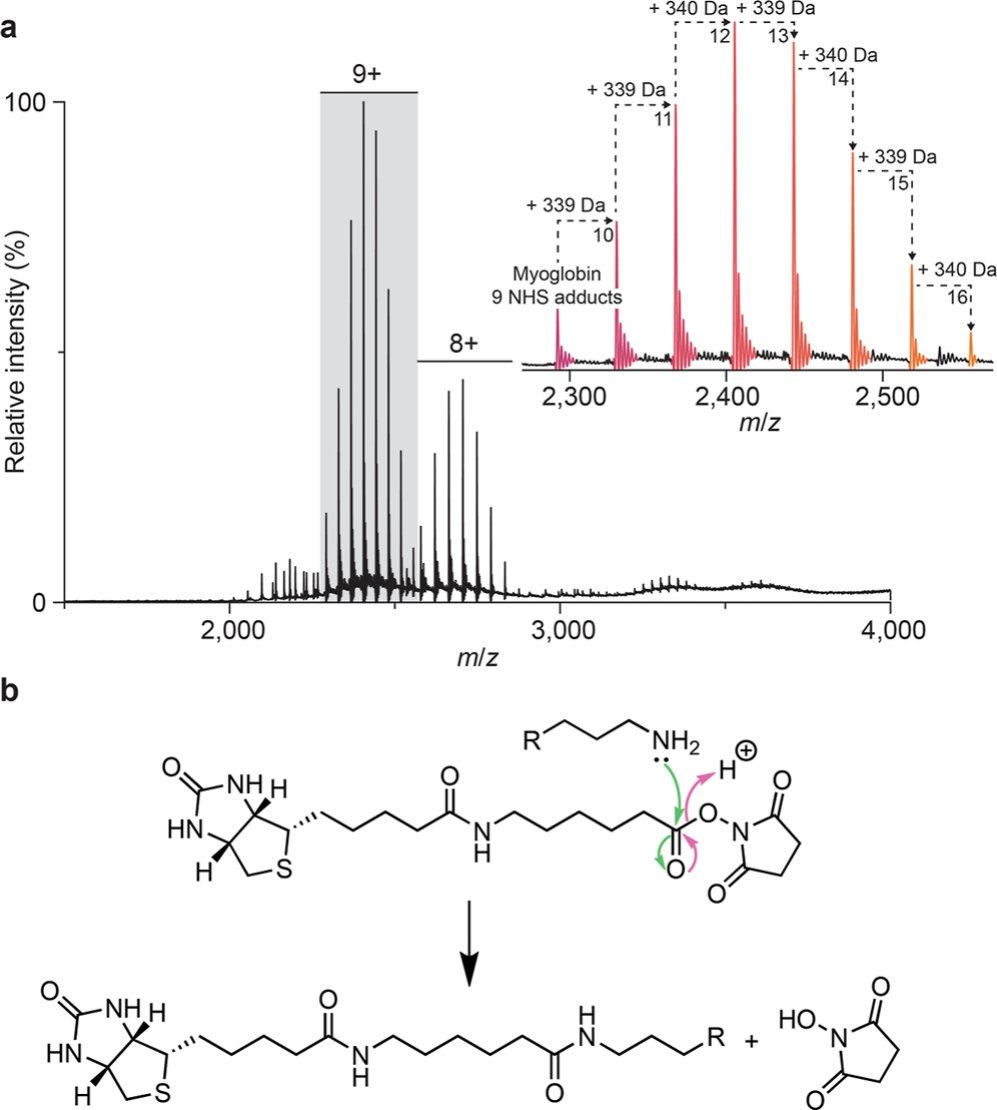
NHS ester labeling of equine myoglobin. (a) Native mass spectrum of equine myoglobin (100 mM ammonium acetate, pH 7.0) following reaction with a 25-fold molar excess of biotinamidohexanoic acid *N*-hydroxysuccinimide (NHS) ester for 30 min at 37 °C. Up to 16 biotin adducts can be observed. (b) Reaction mechanism for NHS ester labeling. The activated ester reacts with primary amines on lysine side chains (and the N-terminal α-amino group) to form a stable amide bond, releasing NHS as the leaving group. Initial nucleophilic attack by the primary amine is shown with green arrows; subsequent elimination of the NHS leaving group is shown with pink arrows.

Integrating across all species, we calculated the mean number of adducts to be 12.02 adducts/protein, below the total number of primary amines theoretically available (19 lysine residues and the N terminus). The adduct-count distribution was compared to Poisson and binomial models as references for stochastic, independent modification (Figure S4). While the overall shape was similar, the experimentally observed distribution was systematically narrower than both, consistent with labeling governed by a finite number of primary amines with heterogeneous reactivities, such that modification becomes progressively self-limiting as the more reactive sites are occupied.

Because the measurement was conducted under nondenaturing conditions, we also confirmed that NHS ester labeling did not disrupt heme binding and did not produce appreciable side reactions (e.g., hydrolyzed biotin-NHS or protein oxidation). In contrast, when the same protein sample was analyzed under denaturing conditions (Figure S5), we observed fewer modifications (mean number of adducts = 10.73) suggesting partial loss of NHS modifications during denaturing preparation and/or analysis. As anticipated, native MS therefore enabled us to robustly characterize the overall composition and stoichiometry of reaction products, comparable to denaturing intact mass measurements, while avoiding associated artifacts.

### Unbiased Evaluation of NHS Ester Chemistry with nTDMS

Intact mass measurements of biotin-conjugated myoglobin revealed the overall composition of the reaction products. However, the site-specific reactivity of individual primary amines remained unresolved. To ascertain residue-level detail, we sequentially isolated each adduct (9+ charge state) and subjected the ions to HCD–a form of beam-type collision-induced dissociation. The potential difference used to accelerate ions into the HCD cell was minimized to limit internal fragment formation and prevent decomposition of modifications during fragmentation. For the species bearing 11 biotin–NHS adducts (Figure 2a), a primary product at *m*/*z* 2586.60 (most-abundant isotopologue) *z* = +8, was observed. This envelope was assigned to *apo-*myoglobin with four biotin–NHS adducts, formed via the loss of a singly charged heme; the neutral mass (20,684.8 Da) closely matches theory (20,683.3 Da). The surrounding spectrum also contained multiple additional envelopes with charge states between 1+ and 8+, consistent with sequence ions produced during top-down fragmentation.

**Figure 2:**
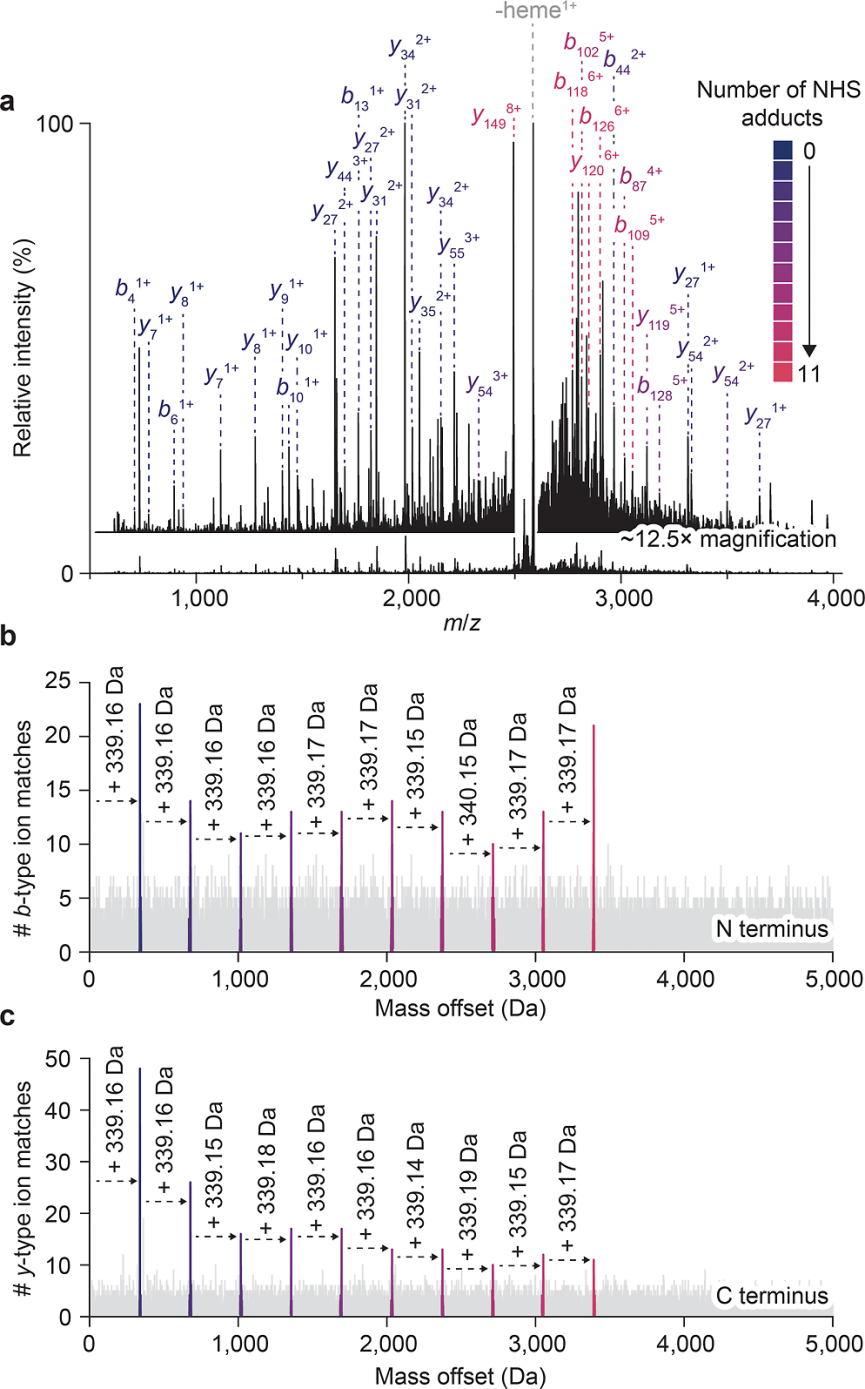
Native top-down mass spectrometry and the fragment-level open search elucidate NHS ester modification patterns. (a) Native top-down mass spectrum of myoglobin bearing 11 biotin–NHS adducts (9+ charge state). The protonated precursor was isolated with the quadrupole and subjected to ion-neutral collisions (HCD 80 V). Fragment ion peaks are annotated, with colors corresponding to the number of biotin–NHS adducts present on each sequence ion. (b, c) Fragment-level open search analysis assigns modification states to individual sequence ions originating from the (b) N-terminal and (c) C-terminal regions. Up to 10 adducts were reliably observed on *b*- and *y-*type sequence ions.

To interpret these fragments, we deconvolved MS^2^ spectra with precisION,^27^ detecting 1000–2000 envelopes after machine-learning–based filtering. We then performed a fragment-level open search, identifying statistically significant sets of fragment ions that share a common mass offset from canonical *b*- and *y*-type sequence ions. This search mode enables modifications of any mass to be detected without prior knowledge or a database. For the 11-adduct myoglobin, the open search confirmed the mass of the modification as 339.1648 Da (3.1 mDa mass error), and enabled independent identification of up to 10 adducts on *b*- and *y*-type ions (Figure 2b,c). For all modification stoichiometries examined (9–14), no other significant mass offsets were detected (aside from sodium adduction, water loss, and monoisotopic errors), indicating that the heme cofactor is completely released during fragmentation, and that the biotin–NHS modification did not decompose upon activation (Figure S6). While side reactivity was not observed here, many electrophiles can react through unanticipated pathways under specific conditions.^44,45^ The fragment-level open search therefore provides a general strategy for characterizing off-target reactions, leveraging the high-accuracy modification masses obtainable from nTDMS to elucidate reaction mechanisms. Moreover, because portions of covalent probes can be partially labile upon activation,^46^ the open search framework facilitates localization of modifications even after partial decomposition of the adduct.

Overall, for the 11-adduct species we obtained a sequence coverage of 74.3% (161 *b*-type ions and 283 *y*-type ions; Figure 2a), providing information spanning the entire length of the protein. Comparable coverage was also observed across other adduct stoichiometries, with the extent of modification having little effect on spectral quality. In contrast, denaturing top-down analysis yielded substantially reduced coverage due to extensive interference (Figure S5). We therefore reasoned that such extensive coverage under native conditions would enable modifications to be localized and quantified with relative precision, despite the apparent promiscuity of the NHS ester.

### Quantitative Mapping of Amine Reactivity

nTDMS can be readily applied to localize modifications when they are primarily located at a single site.^30,47^ This is often the case for targeted covalent probes that react with specific residues (e.g., many activity-based protein profiling reagents^1,2^ and covalent inhibitors^6,29,30^ ). However, many electrophiles–including NHS esters–display broader and more heterogeneous reactivity, complicating site-level localization. To quantify site-specific reactivities with nTDMS for a range of reactivity profiles (Figure S7), we developed a deconvolution framework that addresses two key challenges inherent to such analyses: (i) low signal-to-noise ratios and overlapping isotopic envelopes that can distort individual fragment-ion quantification, and (ii) incomplete fragmentation between (near-)­adjacent reactive residues that limits direct measurement of individual sites.

Briefly, we compiled sequence ions exhibiting multiple modification stoichiometries with consistent charge states and used them to calculate the average number of adducts between each fragmentation site and the N-terminus of the protein (Figures 3a scatter points, S1, S2; see Experimental). We then grouped reactive residues into bins–sets of residues that could not be independently resolved based on the available fragment coverage in all six fragment spectra. Here, given the good sequence coverage, we were able to divide the 20 reactive sites into 15 bins, with [Lys46/48], [Lys63/64], [Lys78/79/80], and [Lys97/99] remaining unresolved. The occupancy of each bin was estimated by constrained least-squares fitting to a step function, with steps allowed at reactive residues (Figures 3a black trace, S8), subject to non-negativity, a maximum step size equal to that of the bin size, and a fixed total number of adducts. This formulation yields a quantitative, stoichiometry-resolved “reactivity map” across the protein sequence, with uncertainties estimated by residual bootstrap resampling (Figures 3b, S9). This approach builds upon prior foundational top-down strategies for measuring site-specific occupancies,^48,49^ while extending the fitting framework to provide more robust error estimates and to accommodate increasingly promiscuous modification behavior e.g., multiple modifications distributed across many (10+) residues.

**Figure 3:**
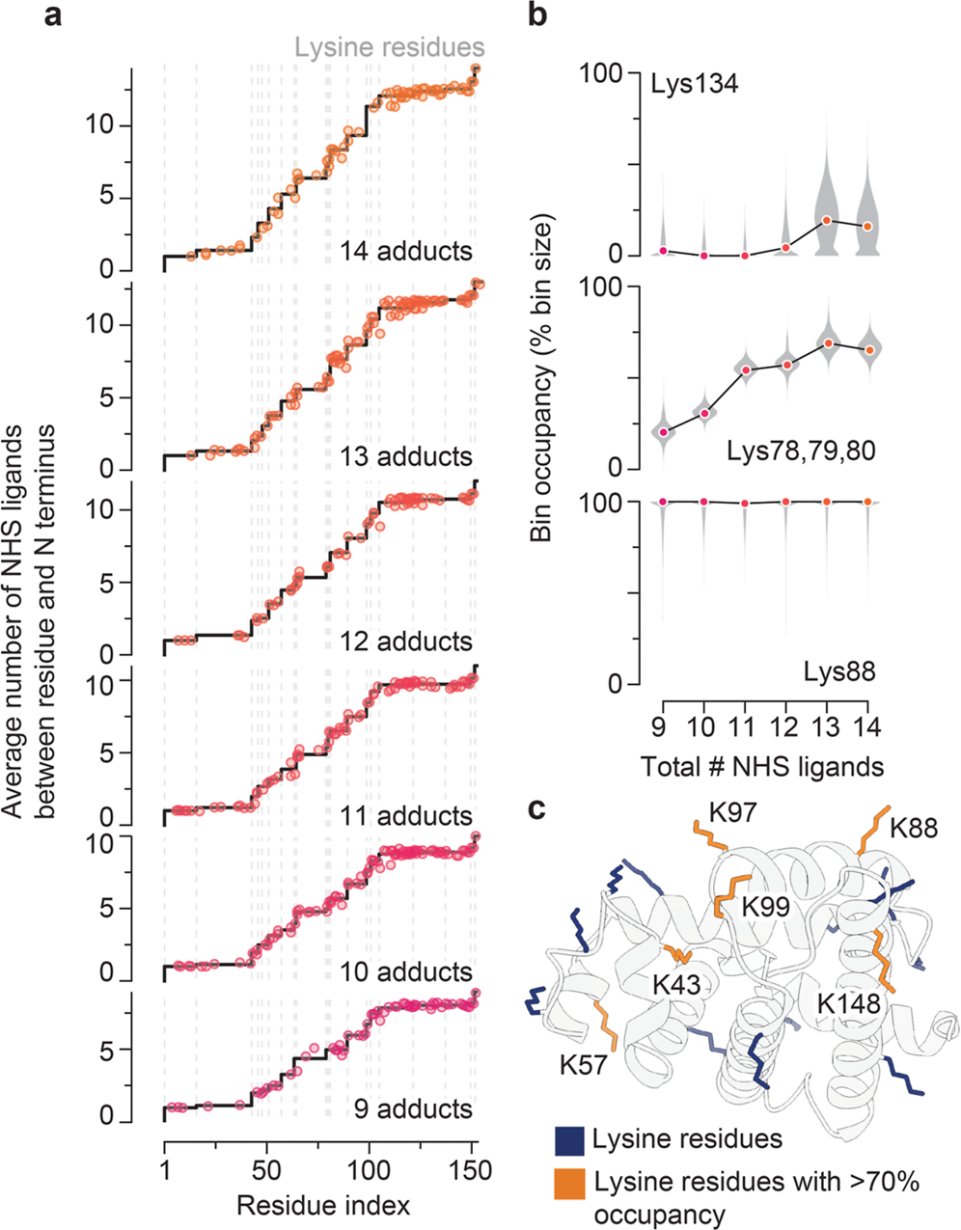
Quantitative mapping of site-specific NHS ester reactivity. (a) Cumulative biotin–NHS labeling profiles for myoglobin bearing 9–14 adducts per protein (bottom to top). Points represent experimentally determined occupancies from *b*- and *y*-type sequence ions. Black step traces show per-primary amine reactivity values obtained by constrained fitting of the experimental data; vertical gray lines indicate lysine positions. (b) Scatter plots illustrating the change in occupancies of selected lysine residues as a function of precursor stoichiometry. Individual sites were generally observed to increase in occupancy with increased precursor stoichiometry. Both high-reactivity (e.g., Lys88) and low-reactivity residues (e.g., Lys134) were observed. Uncertainty distributions derived from residual bootstrap (*n* = 10,000) resampling are displayed as violin plots. (c) AlphaFold database model of equine myoglobin with lysine residues colored by apparent NHS reactivity (orange: >70% occupancy per residue; blue: other lysine residues). Highly reactive lysine residues are labeled.

As expected, for species carrying 9–14 biotin–NHS adducts, the cumulative number of adducts increased with distance from the N terminus (Figures 3a, S8). In all cases, the cumulative adduct count approached that of the intact precursor, confirming minimal modification loss during fragmentation and supporting the quantitative accuracy of our fragment-based analysis. Comparing site-resolved occupancies across the range of precursor stoichiometries, we observed a broad range of reactivities, spanning residues that were rarely modified (e.g., Lys34), to those exhibiting near-complete occupancy across all precursors (e.g., Lys134) (Figures 3b, S9). Taking associated uncertainties into account, site-resolved occupancies were largely consistent across precursor stoichiometries, with a general trend toward increased occupancy with an increased total number of adducts consistent with relatively independent, sequential modification events. Such precursor-stoichiometry–resolved profiles should prove valuable for identifying deviations from this typical noncooperative behavior, including modification crosstalk in biological proteoforms^15^ or cooperative binding by covalent drugs. These effects would be difficult to detect using bulk measurements that report only average occupancies, such as bottom-up proteomics.

To probe individual residue reactivity, we computed the precursor-abundance–weighted mean occupancy for each site, yielding precise estimates of average residue reactivity (Figure S10). To further test whether modification events occur independently, we simulated the expected MS^1^ stoichiometry distribution using the estimated site occupancies as independent modification probabilities. Close agreement between the experimental and simulated distributions (Figure S11) supports the hypothesis that NHS modifications occur as largely independent events, without significant positive or negative cooperativity between sites. Deviations from this agreement (e.g., over- or under-representation of particular stoichiometries) would indicate cooperative effects. The combined reactivity data further revealed the N terminus of the protein to be highly reactive, likely reflecting the distinct p*K*_a_ of its α-amino group.^50^ Furthermore, Lys43, Lys57, Lys88, [Lys97/99] and Lys148 all exhibited average occupancies greater than 70% (Figures 3c, S10). Solvent exposure alone cannot sufficiently account for this increased reactivity, as many of the comparatively unreactive amines are also located on the protein surface. Rather, the most reactive residues are in close three-dimensional proximity (Figure 3c), and this clustering may reflect a shared local microenvironment contributing to their elevated reactivity. We envision that this additional layer of information–that is, the spatial density of adducts on native proteins–will offer valuable insights for applications where covalent ligands are used to elucidate cellular architectures and protein–protein interaction partners.

### nTDMS Provides Greater Certainty and Coverage than Peptide Mapping

To assess the quantitative accuracy of the nTDMS-based approach, we analyzed chymotryptic peptides generated from the same modified myoglobin sample using a conventional bottom-up proteomics workflow (Figure S12). Trypsin was not used for digestion, as modification of lysine residues was expected to compromise its cleavage efficiency and bias quantification. We detected 24 unique peptides from myoglobin at a 1% false-discovery rate. Collectively, these peptides spanned 76% of the protein’s sequence. Notably absent from this data was a continuous 34-residue region containing seven of the 20 potentially reactive residues (Figure S12b). When quantifying individual site-resolved occupancies, we observed close agreement between nTDMS-derived and LFQ-based estimates for several residues (Figure S12c), highlighting the quantitative accuracy of both approaches where sufficient peptide coverage was available. However, for a subset of sites, occupancies could not be confidently determined from the bottom-up data, with large discrepancies observed between estimates derived from distinct peptides. This variability arises because positional isomers often coelute and cannot be reliably distinguished or independently quantified by automated peptide-centric workflows, causing individual peptide measurements to reflect unresolved mixtures of modification states. These limitations reduce confidence in site-specific occupancy estimates for complex modification patterns. Additionally, large and/or charge modifications may alter peptide response factors, introducing further quantitative discrepancies. In contrast, nTDMS provides continuous coverage of the intact proteoform and preserves modification connectivity with minimal artifacts, enabling more complete, confident, and internally consistent quantification of site-resolved reactivity.

## Conclusion

We developed a generalizable nTDMS-based framework that quantitatively maps electrophile reactivity at both the level of the intact protein and at individual sites. Using NHS ester modification of myoglobin as a model system, we demonstrated that site-specific occupancies can be extracted even for promiscuous electrophiles that produce heterogeneous modification patterns. Incorporating a fragment-level open search, the method can be readily extended to diverse electrophilic probes, including those with poorly defined or unanticipated reactivities. By preserving both native protein structure and modification connectivity throughout the analysis, our approach sidesteps the artifacts inherent to denaturation and proteolysis while providing residue-level resolution previously accessible only through bottom-up workflows.

The native conditions employed here open new possibilities beyond simple modification mapping. Further applications could examine how chemical modifications affect biomolecular interactions, or how post-translational modifications modulate electrophile reactivity–mechanistic questions that are difficult to address using conventional denaturing workflows. While current technical limitations result in some quantitative uncertainty (particularly for closely spaced reactive residues), ongoing advances in ion activation methods^51–53^ and emerging technologies like charge-detection mass spectrometry^54–56^ promise to enhance both sequence coverage and fragment ion quantification.

As these technical improvements materialize, we envision this approach will further exceed the quantitative accuracy of flagship bottom-up methods while uniquely preserving proteoform-level information. This capability will prove essential for developing next-generation covalent therapeutics and activity-based probes, where understanding modification patterns at the intact proteoform greatly influences biological outcome.

## Supplementary Material



## Notes

Supplementary note; occupancy plots, full open search results, representative reactivity profiles, denaturing analysis, peptide mapping (PDF) (PDF).
