# Supplementary material for: Quantitative Mapping of NHS Ester–Protein Reactivity Using Native Top-Down Mass Spectrometry
Source: J Am Soc Mass Spectrom. 2026 Jan 28;37(3):702–9. doi: 10.1021/jasms.5c00395 (PMC12964535; doi:10.1021/jasms.5c00395)
Supplement: Supplementary file 1 [file js5c00395_si_001.pdf]

Supporting Information

Quantitative Mapping of NHS Ester–Protein Reactivity Using Native Top-Down Mass Spectrometry

Jack L. Bennett[1,2‡], Olivia B. Ramsay[1,3‡], Corinne A. Lutomski[1,2], Carla Kirschbaum[1,2], and Carol V. Robinson[1,2*]

1. Kavli Institute for Nanoscience Discovery, Dorothy Crowfoot Hodgkin Building, University of Oxford, Oxford OX1 3QU, UK
2. Department of Chemistry, Physical and Theoretical Chemistry Laboratory, University of Oxford, Oxford OX1 3QZ, UK
3. Department of Oncology, Old Road Campus Research Building, University of Oxford, Oxford OX3 7DQ, UK

‡ These authors contributed equally.

* Corresponding author: carol.robinson@chem.ox.ac.uk

Table of Contents

Figure S1. Stacked bar plots showing the distribution of biotin–NHS modifications 2

Figure S2. Unified fitting of *b*- and *y*-type sequence ions 3

Figure S3. Time-dependent modification of equine myoglobin with biotin–NHS 4

Figure S4. Modification of myoglobin appears underdispersed relative to an ideal process 5

Figure S5. Denaturing myoglobin for top-down analysis 6

Figure S6. Identification of biotin–NHS modifications across precursor stoichiometries 7

Figure S7. Possible patterns of covalent probe reactivity 8

Figure S8. Stoichiometry-resolved modeling of site-specific NHS reactivity 8

Figure S9. Stoichiometry-resolved NHS occupancy profiles 9

Figure S10. Average occupancies of reactive residues 10

Figure S11. Occupancies enable accurate modeling of the precursor distribution 10

Figure S12. Peptide mapping by bottom-up proteomics is consistent with nTDMS 11

Supplementary Note 1. Residue occupancy model 12

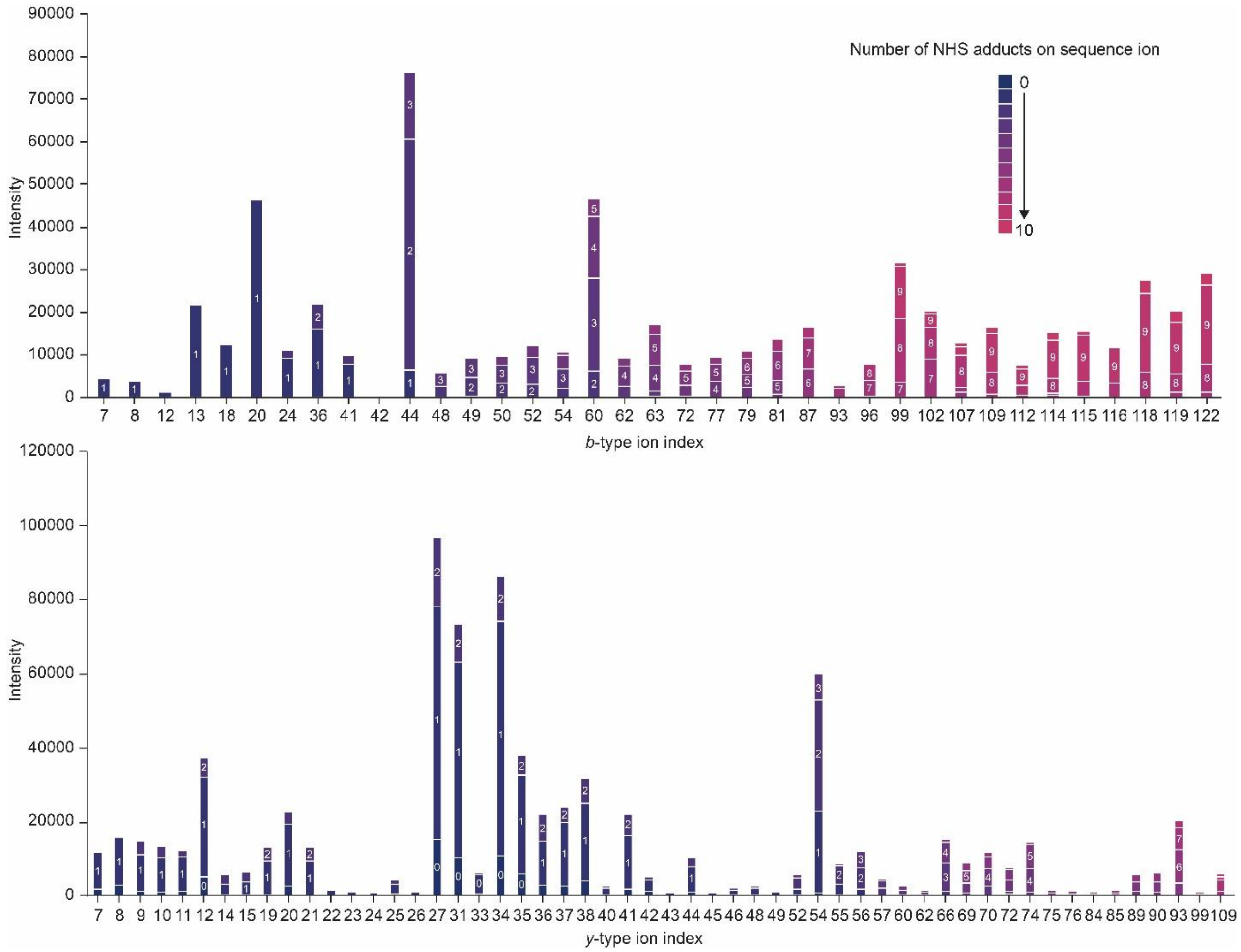

Figure S1. Stacked bar plots showing the distribution of biotin–NHS modifications across myoglobin *b*- and *y*-type sequence ions. Raw intensities of differentially modified sequence ions produced from myoglobin with ten biotin–NHS adducts are shown for *b*-type (top) and *y*-type (bottom) ions. Intensities are normalized by charge state to account for charge-dependent signals in Orbitrap mass spectra. Sequence ions with different numbers of biotin–NHS adducts are indicated by distinct colors and labelled where possible.

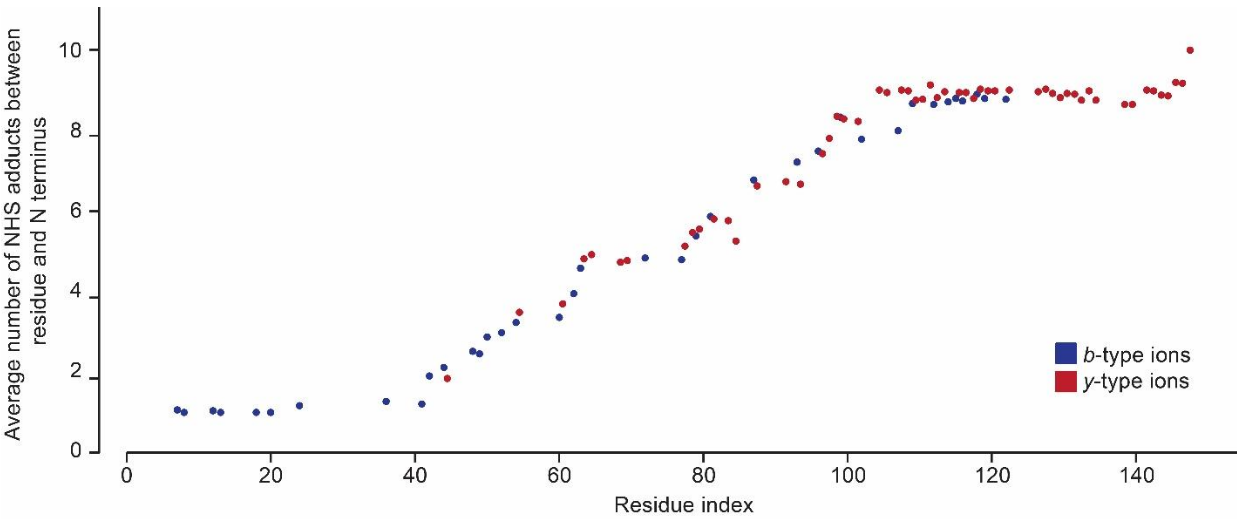

Figure S2. Unified fitting of *b*- and *y*-type sequence ions. Intensities from *y*-type ions are transformed into an N-terminal reference frame using the procedure described in **Supplementary Note 1**, enabling joint analysis with the *b*-type ions.

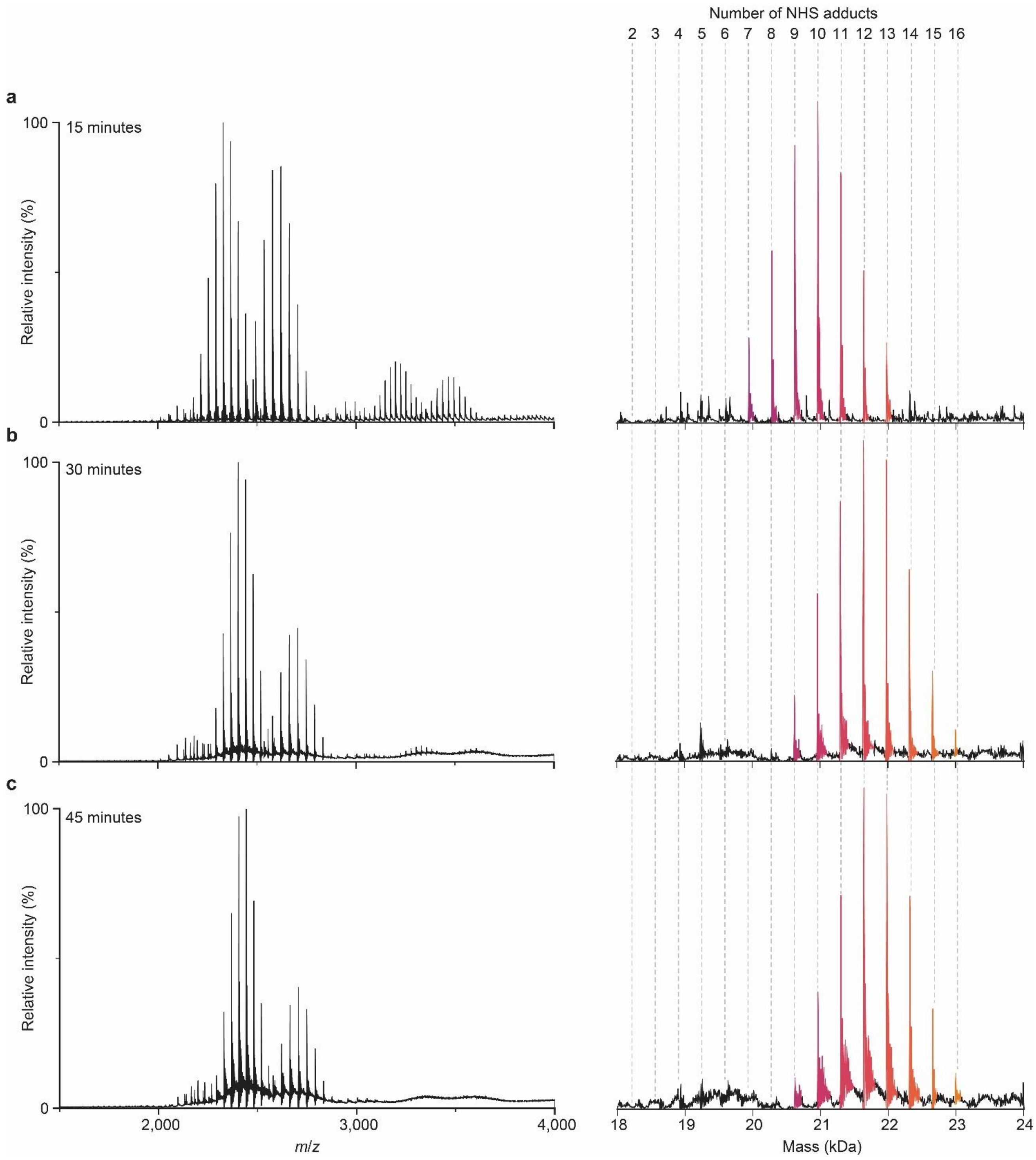

Figure S3. Time-dependent modification of equine myoglobin with biotin–NHS. Native mass spectra (left) and corresponding deconvolved mass-domain spectra (right) of myoglobin in 200 mM ammonium acetate (pH 7.0) after reaction for (a) 15 min, (b) 30 min, and (c) 45 min with a 25-fold molar excess of biotin–NHS. The extent of modification reaches a plateau after approximately 30 min. The minor distribution between m/z 3,000–3,700 was assigned to non-specific myoglobin dimers.

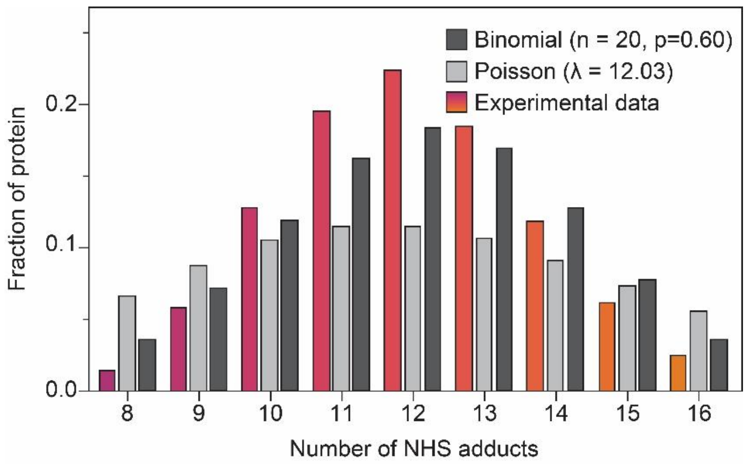

Figure S4. NHS ester modification of myoglobin appears underdispersed relative to an ideal Poisson process. The observed distribution of biotin–NHS adduct stoichiometries is compared with Poisson and binomial distributions used as references for stochastic modification. The lower observed variance suggests that labeling is constrained relative to a purely stochastic process with heterogenous reactivities.

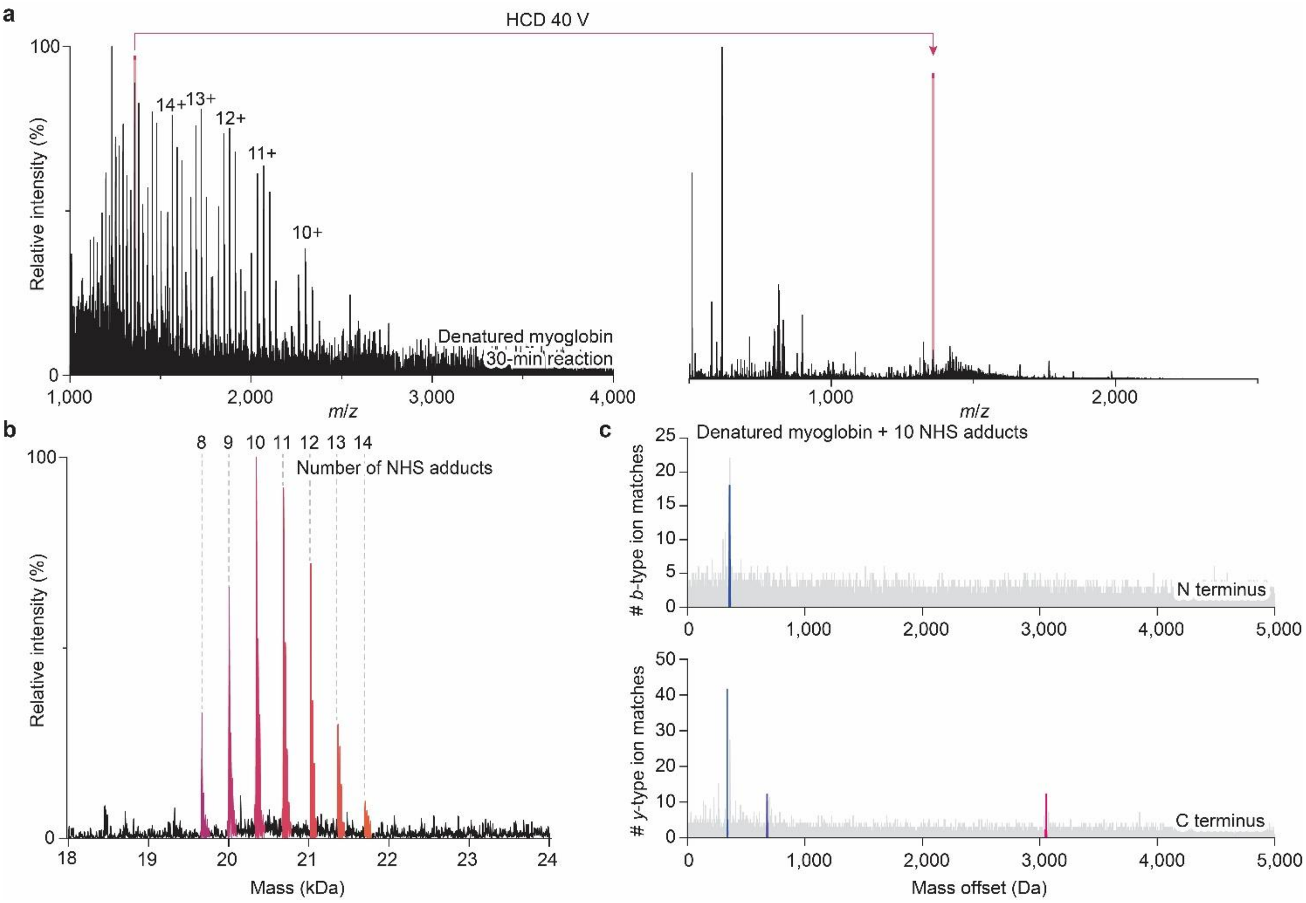

Figure S5. Denaturing myoglobin for top-down analysis provides reduced information on NHS modification and introduces experimental artifacts. (a) Mass spectrum of myoglobin in a solution of 200 mM ammonium acetate (pH 7.0), acetonitrile, and isopropanol (50:45:5 (v/v)) supplemented with 1% formic acid, following a 30-min reaction with biotin–NHS. *apo*-Myoglobin bearing 10 NHS adducts (15+ charge state) was quadrupole-isolated and activated using HCD (40 V) to generate a high-quality fragment spectrum (right panel). (b) Deconvolved mass-domain spectrum derived from the denaturing analysis. Fewer NHS adducts were observed than under native conditions, suggesting adduct dissociation. (c) Fragment-level open search results for *apo*-myoglobin with 10 NHS adducts. In contrast to native analyses, very few significant offsets can be observed for either *b*-type (upper) or *y*-type ions (lower).

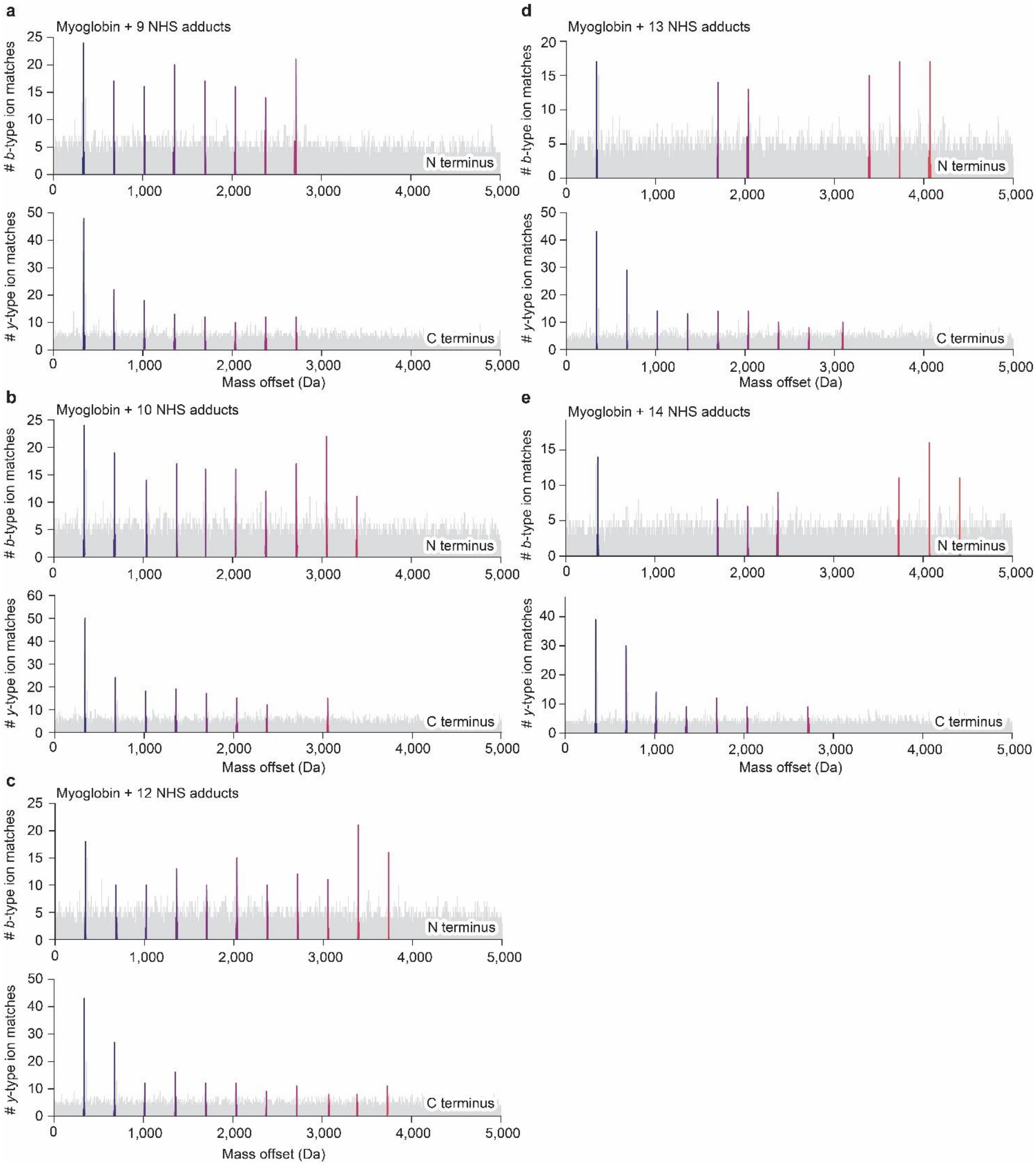

Figure S6. Identification of biotin–NHS modifications across precursor stoichiometries. Fragment-level open search results are shown for precursors containing (a) 9, (b) 10, (c) 11, (d) 13, and (e) 14 biotin–NHS adducts. N- (upper) and C-terminal (lower) mass offset scans are shown for each species. In each spectrum, peaks corresponding to fragment ions with the expected number of adducts are readily observed.

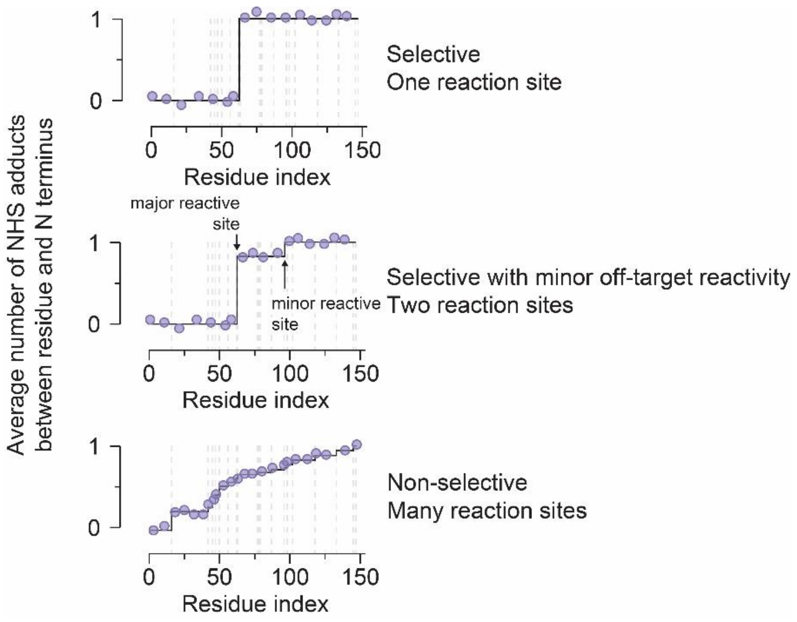

Figure S7. Possible patterns of covalent probe reactivity. Simulated modification stoichiometry profiles are shown for a highly selective inhibitor (top), a mostly selective inhibitor with minor off-target reactivity (middle), and a broadly reactive, non-selective electrophile (bottom). Data were simulated assuming a precursor with a single modification was isolated and activated.

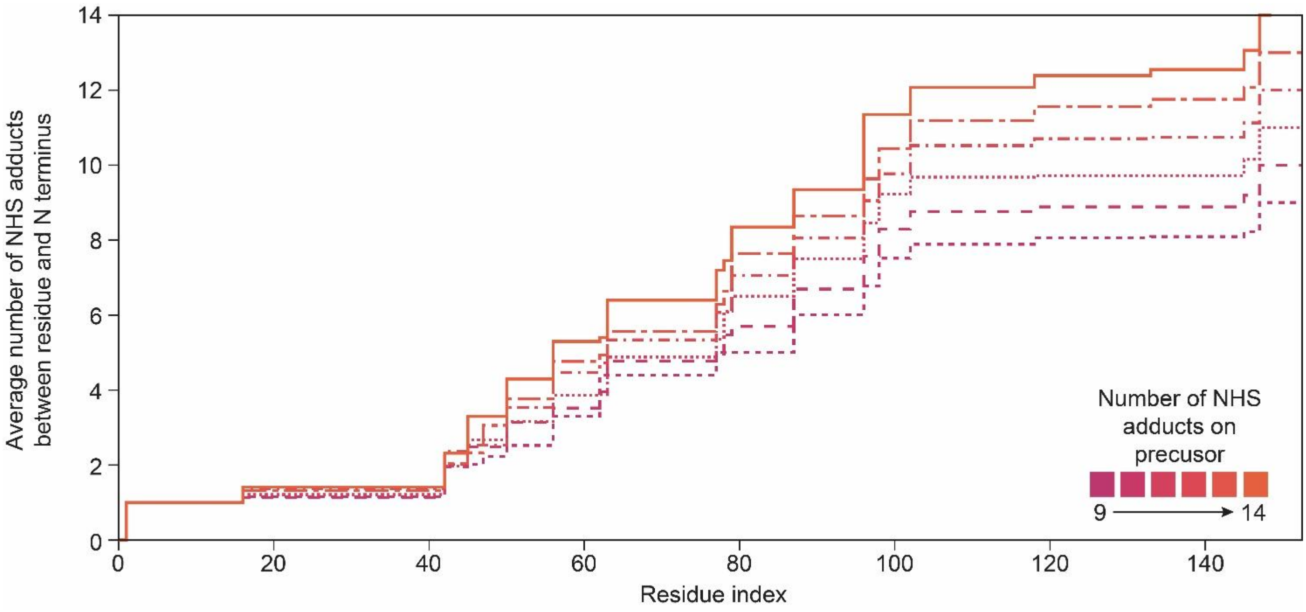

Figure S8. Stoichiometry-resolved modeling of site-specific NHS reactivity. Overlaid biotin–NHS labeling profiles for myoglobin bearing 9–14 adducts per protein. Step functions were derived from least-squares fitting to the experimentally-derived average number of NHS adducts between residues and the N terminus.

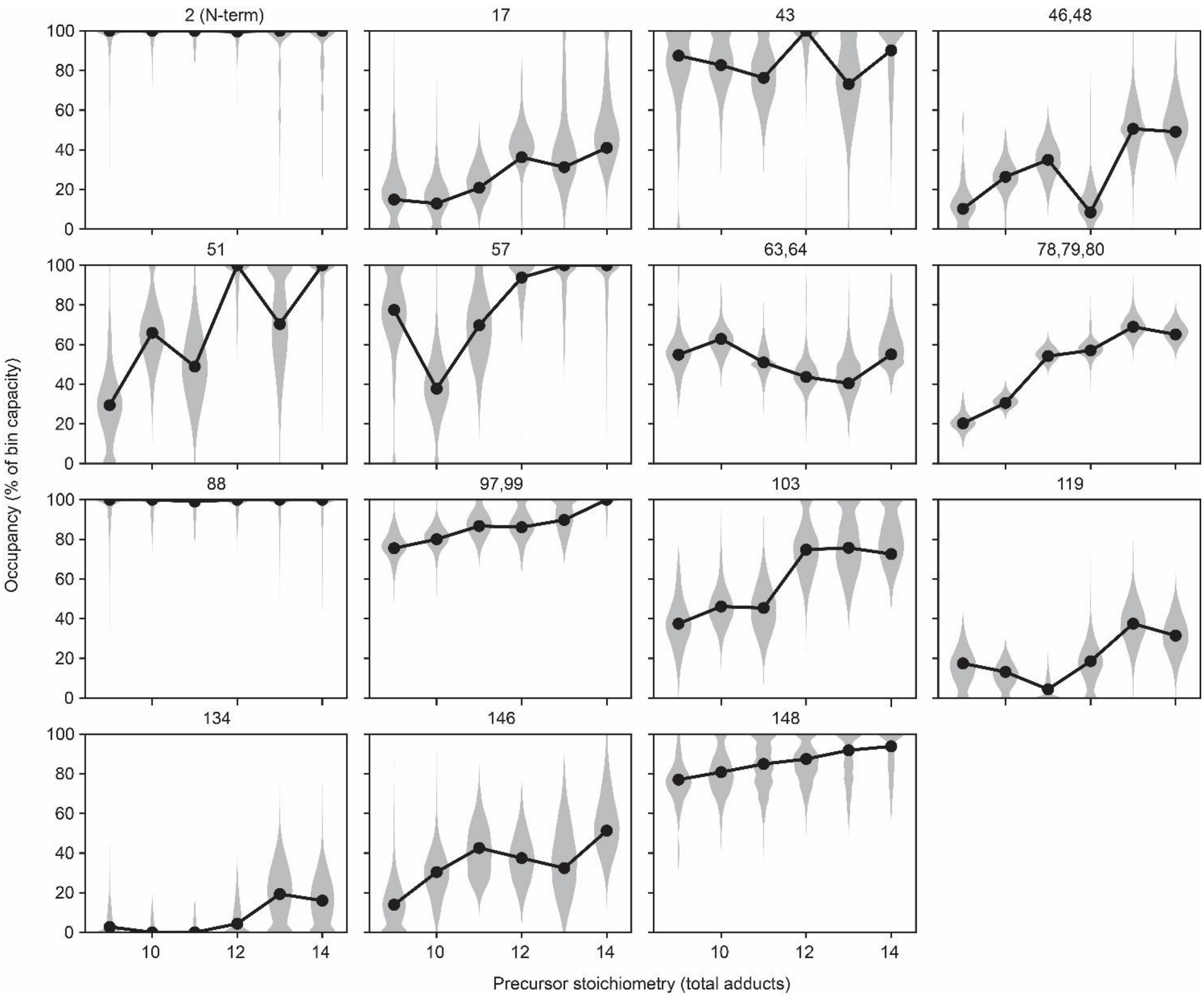

Figure S9. Stoichiometry-resolved NHS occupancy profiles. Individual subplots show the fractional occupancy of reactive residue bins as a function of the total number of NHS adducts on the precursor. Residues included in each bin are listed above each plot and numbered according to the unprocessed protein sequence. Values obtained from fitting are shown as black points. Uncertainty distributions were derived from residual bootstrap resampling (n = 10,000) and are displayed as violin plots.

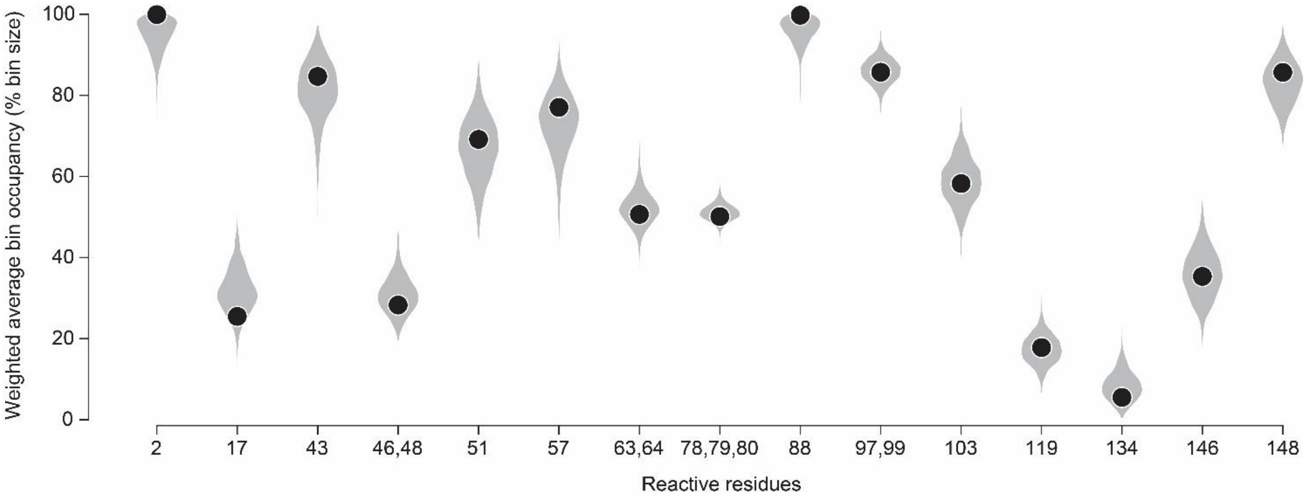

Figure S10. Average occupancies of reactive residues. Plot showing the abundance-weighted mean fractional occupancy of reactive residue bins across all examined precursor stoichiometries. Point estimates were scaled by the relative abundance of each precursor population. Uncertainty distributions were obtained by propagating residual-bootstrap samples through the abundance-weighted average.

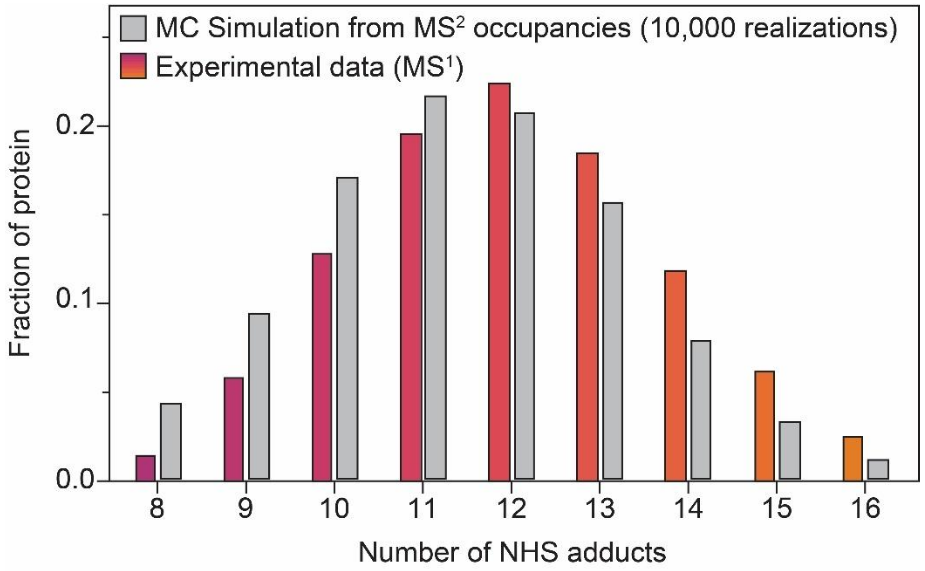

Figure S11. MS^2-derived occupancies enable accurate modeling of the precursor distribution. The experimentally observed distribution of NHS adduct stoichiometries (colored) was compared to the results from a Monte Carlo simulation (10,000 realizations; grey). In each realization, the number of modifications in each reactive residue bin was randomly sampled from a binomial distribution parameterized by the experimentally determined mean occupancy and finite site capacity of that bin.

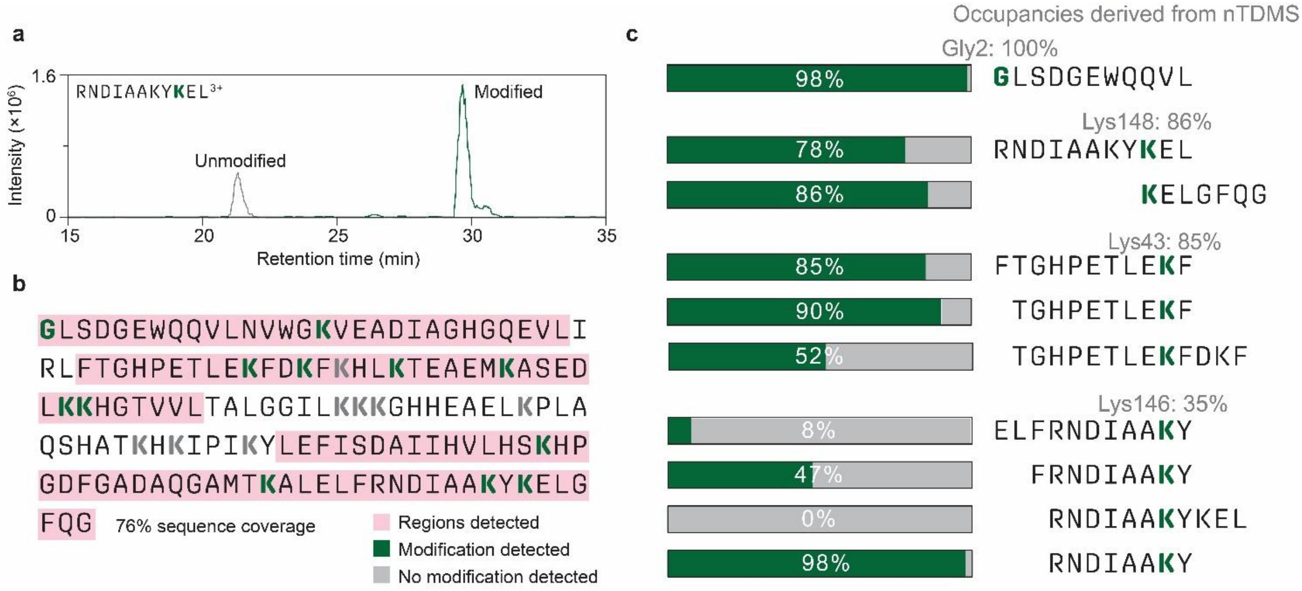

Figure S12. Peptide mapping by bottom-up proteomics is consistent with nTDMS but provides reduced insight into site-resolved occupancies. (a) Extracted ion chromatograms (XICs) for a representative modified peptide, RNDIAAKYKEL (3+), identified from a chymotryptic digest of the same myoglobin sample used for nTDMS analysis (30-min reaction). Both unmodified (gray) and NHS-conjugated (green) forms were identified and quantified. Two partially resolved peaks are observed in the XIC for the modified form; these may correspond to positional isomers, but automated analysis did not quantify the alternate isomer. (b) Sequence map showing regions of myoglobin detected by peptide mapping. Peptides spanning a large central segment of the protein were not identified. (c) Site-specific occupancies derived from label-free quantification. Bar charts show the occupancy of individual reactive residues. Peptides used for quantification are displayed to the right, with reactive residues highlighted in green. Occupancies derived from nTDMS are shown above each site in gray.

Supplementary Note 1. Residue occupancy model

Data and notation. Let the protein have length L residues and let $\mathcal{C} = \{c_i\}_{i=1}^{M}$ denote the set of observed backbone cleavages, indexed from the N terminus. For each cleavage c_i, we measure an estimate $\mu_i \in [0, T]$ of the cumulative adduct count between the N terminus and c_i, where T is the total number of adducts observed on the intact precursor.

Conversion of *y*-type ions to the N-terminal reference frame. *y*-type ions arising from cleavage at c_i report cumulative adducts from the C terminus, $\mu_k^{(C)}$, where $i + k = L$. These estimates are accordingly converted to the N-terminal frame by

$$\mu_i^{(N)} = T - \mu_{L-i}^{(C)}$$

After this transformation, we can perform a joint fit to data from both *b*- and *y*-type sequence ions.

Construction of modification site bins. Let $\mathcal{K} = \{k_1, k_2, \ldots, k_R\}$ denote the indices of primary amines that may be modified. Some primary amines cannot be distinguished because no cleavage falls between them i.e., $c_i < k_m < k_{m+1} < c_{i+1}$. We therefore partition $\mathcal{K}$ into B resolvable bins:

$$\mathcal{K} = \bigcup_{j=1}^{B} \mathcal{S}_j$$

where each bin $\mathcal{S}_j$ contains one or more primary amines that are indistinguishable given the available fragment coverage. For each bin we define a representative residue index

$$r_j = \min(\mathcal{S}_j)$$

Occupancies. We parameterize the modification pattern by the per-bin occupancy vector

$$\boldsymbol{p} = (p_1, p_2, \ldots, p_B)^{\top}$$

where $p_j \geq 0$ is the combined number of adducts per protein assigned to residues in bin j.

Cumulative ladder matrix. We define a binary matrix $A \in \{0,1\}^{M \times B}$ by

$$A_{ij} = \begin{cases} 1, & \text{if } r_j \leq c_i \\ 0, & \text{otherwise} \end{cases}$$

This matrix encodes the fact that the cumulative adduct count μ_i at cleavage c_i equals the sum of occupancies of all bins located N-terminal to that cleavage site.

Thus, the model prediction at cleavage c_i is:

$$\hat{\mu}_i = \sum_{j=1}^{B} A_{ij} p_j$$

or in vector form:

$$\hat{\boldsymbol{\mu}} = A\boldsymbol{p}$$

where $\boldsymbol{\mu} = (\mu_1, \mu_2, \ldots, \mu_M)^\top$ and $\hat{\boldsymbol{\mu}} = (\hat{\mu}_1, \hat{\mu}_2, \ldots, \hat{\mu}_M)^\top$.

Constraints and estimation. Isolating a single precursor ensures that the adduct counts satisfy

$$\sum_{j=1}^{B} p_j = \mathrm{T}, \quad p_j \geq 0 \ \forall j$$

We estimate the per-bin occupancies by solving the constrained least-squares problem

$$\hat{\boldsymbol{p}} = \arg\min_{\boldsymbol{p}} \|A\boldsymbol{p} - \boldsymbol{\mu}\|_2^2 \quad \text{subject to} \ \sum_{j=1}^{B} p_j = \mathrm{T}, \ p_j \geq 0 \ \forall j$$

In practice, optimization is performed using the Sequential Least SQuares Programming algorithm. This procedure yields the set of bin occupancies that best reproduces the combined experimental data.

Uncertainty estimation. After fitting, residuals are computed as

$$\mathbf{r} = \boldsymbol{\mu} - A\hat{\boldsymbol{p}}$$

and centered to zero mean. Bootstrap samples are generated by resampling residuals with replacement to generate synthetic data

$$\boldsymbol{\mu}^{(b)} = \boldsymbol{A}\hat{\boldsymbol{p}} + \mathbf{r} *^{(b)}$$

We then refitted the synthetic dataset for 10,000 replicates

$$\hat{p}^{(b)} = \arg\min_{\boldsymbol{p}} \left\|A\boldsymbol{p} - \boldsymbol{\mu}^{(\boldsymbol{b})}\right\|_2^2 \quad \text{subject to} \ \sum_{j=1}^{B} p_j = \mathrm{T}, \ p_j \geq 0 \ \forall j$$

to yield $\{\hat{p}^{(b)}\}$ which can be used to quantify the uncertainties in per-bin occupancies.
